# Induced Pluripotent Stem Cell-Derived Monocytes/Macrophages in Autoinflammatory Diseases

**DOI:** 10.3389/fimmu.2022.870535

**Published:** 2022-05-06

**Authors:** Takayuki Tanaka, Takeshi Shiba, Yoshitaka Honda, Kazushi Izawa, Takahiro Yasumi, Megumu K. Saito, Ryuta Nishikomori

**Affiliations:** ^1^ Department of Pediatrics, Kyoto University Graduate School of Medicine, Kyoto, Japan; ^2^ Department of Pediatrics, Japanese Red Cross Otsu Hospital, Otsu, Japan; ^3^ Laboratory of Lymphocyte Activation and Susceptibility to EBV Infection, INSERM UMR 1163, Imagine Institute, Paris, France; ^4^ Institute for the Advanced Study of Human Biology (ASHBi), Kyoto University, Kyoto, Japan; ^5^ Department of Immunology, Graduate School of Medicine, Kyoto University, Kyoto, Japan; ^6^ Department of Clinical Application, Center for iPS Cell Research and Application, Kyoto University, Kyoto, Japan; ^7^ Department of Pediatrics and Child Health, Kurume University School of Medicine, Kurume, Japan

**Keywords:** autoinflammatory diseases, induced pluripotent stem cells, disease modeling, drug screening, monocytes, macrophages

## Abstract

The concept of autoinflammation, first proposed in 1999, refers to a seemingly unprovoked episode of sterile inflammation manifesting as unexplained fever, skin rashes, and arthralgia. Autoinflammatory diseases are caused mainly by hereditary abnormalities of innate immunity, without the production of autoantibodies or autoreactive T cells. The revolutionary discovery of induced pluripotent stem cells (iPSCs), whereby a patient’s somatic cells can be reprogrammed into an embryonic pluripotent state by forced expression of a defined set of transcription factors, has the transformative potential to enable *in vitro* disease modeling and drug candidate screening, as well as to provide a resource for cell replacement therapy. Recent reports demonstrate that recapitulating a disease phenotype *in vitro* is feasible for numerous monogenic diseases, including autoinflammatory diseases. In this review, we provide a comprehensive overview of current advances in research into autoinflammatory diseases involving iPSC-derived monocytes/macrophages. This review may aid in the planning of new studies of autoinflammatory diseases.

## Introduction

The concept of autoinflammation, which was first proposed in 1999, refers to seemingly unprovoked and episodic sterile inflammation manifesting as unexplained fever, skin rashes, and arthralgia (1). Autoinflammatory diseases are caused mainly by hereditary abnormalities of innate immunity, without the production of autoantibodies or autoreactive T cells. Analysis of blood cells from patients with autoinflammatory diseases has expanded our understanding of these conditions; however, there are several limitations to this approach: i) collecting enough patient blood samples for analysis is difficult because autoinflammatory diseases are rare and, to make matters worse, many patients are infants, and ii) the *in vitro* phenotype of hematopoietic cells in these patients is affected by existing inflammation or by the prescribed drugs.

The revolutionary discovery of induced pluripotent stem cells (iPSCs), whereby a patient’s somatic cells can be reprogrammed into an embryonic pluripotent state by forced expression of a defined set of transcription factors (2, 3), has the transformative potential to enable *in vitro* disease modeling and drug candidate screening, as well as to provide a resource for cell replacement therapy. iPSC-derived monocytes/macrophages provide an opportunity to analyze the effect of genetic variants in the absence of the limitations described above. Recent reports demonstrate that recapitulating a disease phenotype *in vitro* is feasible for numerous monogenic diseases, including autoinflammatory diseases (4–16).

One of the main obstacles to disease studies based on iPSCs is that directed differentiation of iPSCs is time- and labor-intensive, and the results of functional analysis usually show high variation (even among iPSC clones). To overcome these issues and to obtain a stable and scalable number of mature monocytic cells from iPSC clones, immortalized proliferating myeloid cell lines have been utilized (6, 10). Recent advances in genome editing technology, such as the CRISPR system (17), facilitate functional comparisons between isogenic pairs of mutant and control iPSC clones.

In this review, we provide a comprehensive overview of current advances in research into the role of iPSC-derived monocytes/macrophages in autoinflammatory diseases. We will outline how iPSC-derived blood cells contribute to i) elucidation of disease pathogenesis, ii) functional analyses to facilitate correct diagnosis, iii) evaluation of the disease relevancies of newly identified mutations, and iv) discovery of new drug candidates.

## Advantages and Characteristics of Pluripotent Stem Cell-Derived Macrophages

### Monocyte-Derived Macrophages and Immortalized Cell Lines

**Figure 1:**
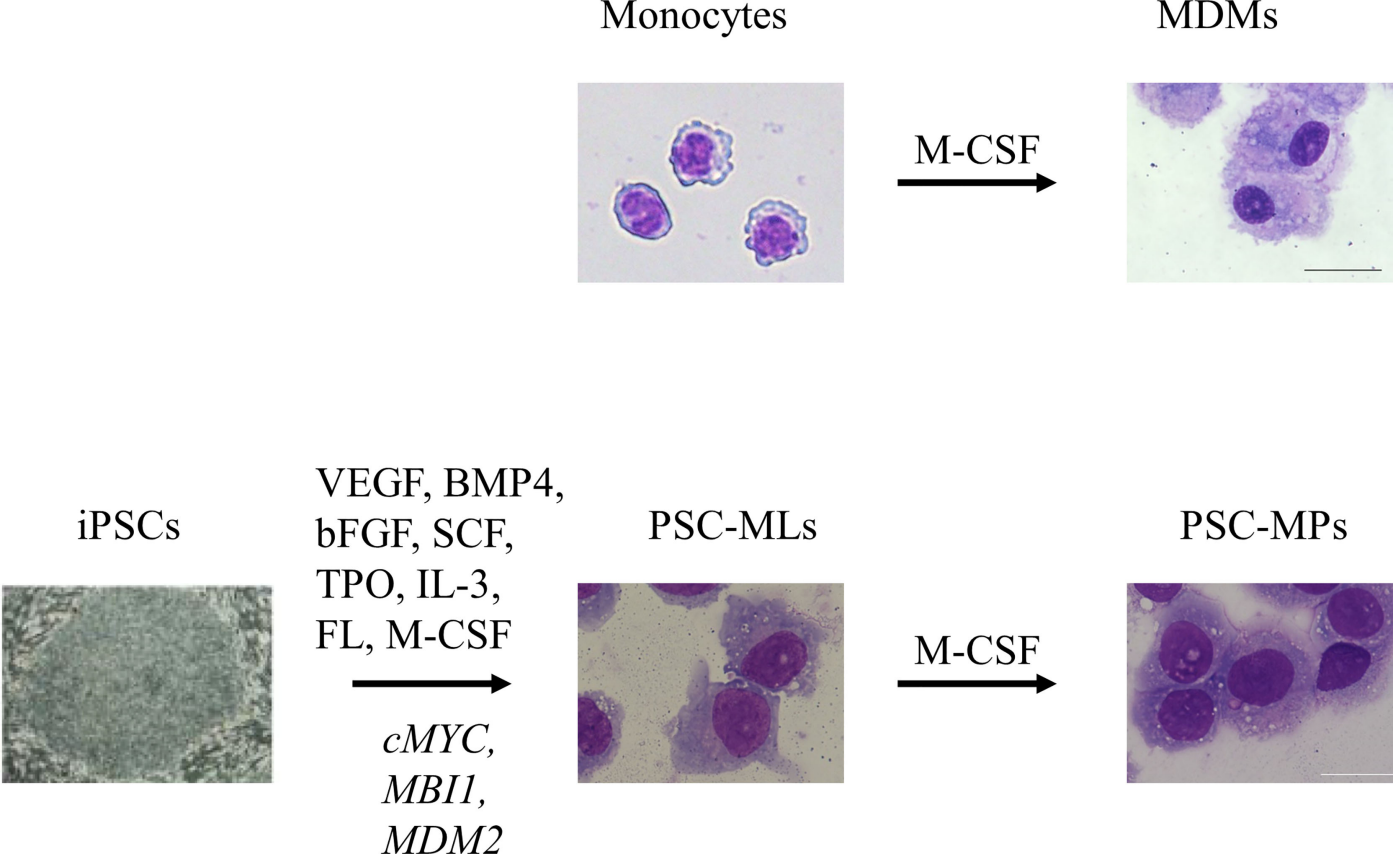
*In vitro* culture of primary monocytes for 7 days in the presence of macrophage colony-stimulating factor (M-CSF) gives rise to adherent monocyte-derived macrophages (MDMs). Sequential stimulation with VEGF, BMP4, bFGF, SCF, TPO, IL-3, FL, and M-CSF differentiates induced pluripotent stem cells (iPSCs) into floating monocyte-like cells. After introduction of three transgenes, namely, *cMYC*, *MBI1*, and *MDM2*, into the floating cells, PSC-derived immortalized myeloid cell lines (PSC-MLs) begin to proliferate. *In vitro* culture of PSC-MLs for 7 days in the presence of M-CSF gives rise to adherent PSC-derived macrophages (PSC-MPs). Scale bars, 20 µm. VEGF, vascular endothelial growth factor; BMP4, bone morphogenic protein type 4; bFGF, basic fibroblast growth factor; SCF, stem cell factor; TPO, thrombopoietin; IL-3, interleukin-3; FL, FLT3 ligand.

The availability of tissue-resident macrophages isolated directly from human tissues is limited due to ethical issues; therefore, monocyte-derived macrophages (MDMs) are used widely for research into human macrophages. This approach involves isolating CD14+ monocytes from peripheral blood mononuclear cells (PBMCs) and exposing them to macrophage colony-stimulating factor (M-CSF) to induce differentiation into macrophages (**Figure 1**) (18). An advantage of the MDM model is that human peripheral blood samples can be obtained without an invasive procedure; indeed, several million monocytes can be obtained from a single venipuncture. Because the *in vitro* culture period is only 1 week, MDMs are most likely free from artifacts that appear after long-term culture; therefore, they should be more representative of the patients’ macrophages. However, MDMs have drawbacks, including limited proliferative capacity and a short culture period, which limit the options when it comes to genetic modification. In an era when pluripotent stem cell (PSC)-derived macrophages (PSC-MPs) are available, MDMs remain an important research tool for autoinflammatory diseases because they are likely more “physiological” than PSC-MPs. For example, MDMs have been used to examine the clinical relevance of findings obtained using PSC-MPs (6) and to examine functional differences between primary monocytes and macrophages with respect to cytokine secretion (12) (see below).

The immortalized cell lines THP-1 and U937 are used as alternative sources of macrophages because they expand spontaneously and are amenable to genetic manipulation. These cell lines originate from the peripheral blood of patients with acute monocytic leukemia and contain highly proliferative floating CD14+ “monocyte-like” cells that can differentiate into “macrophage-like” cells upon culture in the presence of phorbol myristate acetate or M-CSF (19). In the field of autoinflammatory disease research, THP-1 cells are used to analyze cell death caused by the expression of mutant *NLRP3* (20, 21). However, as these cells are derived from malignant tumor cells, their biological relevance to non-malignant monocytes/macrophages is limited (22). For example, THP-1 cells secrete only small amounts of cytokines in response to lipopolysaccharide (LPS) stimulation (22), and there are no reports describing increased inflammasome activation in these immortalized cell lines.

### Pluripotent Stem Cell-Derived Macrophages

To overcome the limitations described above, several methods have been developed to generate macrophages from PSCs. In this approach, the culture conditions drive embryonic stem cells or iPSCs to differentiate through a pathway that recapitulates embryonic hematopoiesis (23–26). The advantages of PSC-MPs include easy availability, scalability, standardizability, and easy genetic manipulation (27, 28).

While a few reports have described protocols for differentiating PSCs into monocytes (29), more research has been performed with PSC-MPs than with PSC-derived monocytes, particularly within the field of autoinflammatory diseases (**Table 1**). Many studies report that PSC-MPs and MDMs have similar phenotypes, functions, and transcriptomes (26, 30–33). However, stable differences between PSC-MPs and MDMs were also identified; indeed, it is suggested that PSC-MPs recapitulate embryonic-origin macrophages rather than MDMs, which are derived from definitive hematopoiesis (23–26). Therefore, even though PSC-MPs have been applied successfully to the functional analysis of macrophages in the context of many diseases (**Table 1**), we need to keep in mind that the phenotype of PSC-MPs sometimes differs from that of MDMs or tissue-resident macrophages; where necessary, the validity of findings based on PSC-MPs should be confirmed using MDMs.

**Table 1:** Disease modeling and application of iPSCs to autoinflammatory diseases.

Primary objective	Gene	Disease	Target cell type	Reference
Disease modeling	*NLRP3*	CINCA syndrome	Macrophages, chondrocytes	(4, 5)
	*NOD2*	Blau syndrome	Macrophages	(6, 7)
	*PSMB8*	Nakajo–Nishimura syndrome	Myeloid cell lines	(8)
	*IL-10RB*	Inflammatory bowel diseases	Macrophages	(9)
Diagnosis	*NLRC4*	CINCA syndrome	Myeloid cell lines	(10)
	*NEMO*	Immunodeficiency without obvious ectodermal dysplasia	Myeloid cell lines	(11)
	*MEFV*	Familial Mediterranean fever	Macrophages	(12)
Disease relevancies	*OAS1*	OPAID	Macrophages	(13)
	*NFKB1A*	Autoinflammation with immunodeficiencies	Macrophages	(14)
Drug screening	*NLRP3*	CINCA syndrome	Macrophages	(15)
	*PSMB8*	Nakajo–Nishimura syndrome	Myeloid cell lines	(16)

CINCA, chronic infantile neurologic cutaneous and articular; OPAID, OAS1-associated polymorphic autoinflammatory immunodeficiency.

In early studies, whenever we needed PSC-MPs, we repeated the whole process of differentiating PSCs into macrophages; this process took almost 1 month to generate enough cells for our experiments (4). The differentiation efficiency was not consistent, and the protocol was laborious. To improve the efficiency of differentiation and to standardize macrophage products, we developed a method of cryopreserving PSC-MP (34). For this purpose, we established PSC-derived immortalized myeloid cell lines (PSC-MLs) by introducing *MYC*, *BMI1*, and *MDM2* into iPSC-derived floating monocytic cells (**Figure 1**) (6, 10). The resulting PSC-MLs proliferated vigorously and continuously and were amenable to freeze-and-thaw cycles. After a 1-week culture in the presence of M-CSF, PSC-MLs differentiated into adherent macrophages. Both PSC-MLs and PSC-MPs expressed monocyte/macrophage markers CD45, CD11b, and CD14 and secreted cytokines in response to various stimuli. While immature PSC-MLs were more proliferative than PSC-MPs, terminally differentiated PSC-MPs secreted higher levels of cytokines than PSC-MLs. Therefore, we used both PSC-MLs and PSC-MPs for our research, depending on the goal of each experiment (4, 6–8, 10–12, 15, 16) (**Table 1**). Next, we will outline how PSC-derived blood cells contribute to i) elucidation of disease pathogenesis, ii) functional analysis to facilitate correct diagnosis, iii) evaluation of the disease relevancies of newly identified mutations, and iv) discovery of new drug candidates.

## Elucidation of the Disease Pathogenesis

### Chronic Infantile Neurologic Cutaneous and Articular Syndrome

Chronic infantile neurologic cutaneous and articular (CINCA; MIM 607115) syndrome, also known as a neonatal-onset multisystem inflammatory disease (NOMID), is an autoinflammatory syndrome characterized by systemic inflammation accompanied by an urticarial rash, neurologic manifestations, and arthropathy that begins during the neonatal period (35, 36); it is the most severe form of the autoinflammatory spectrum called cryopyrin-associated periodic fever syndrome. Patients carry a heterozygous gain-of-function mutation in *NLRP3* gene and present with systemic inflammation due to excessive IL-1β production caused by hyperactivation of the NLRP3 inflammasome (37, 38). While approximately half of CINCA patients carry heterozygous gain-of-function mutations in *NLRP3* gene (39), 30%–40% harbor *NLRP3* mutations in only a small number of somatic cells (4.2%–35.8% of blood cells) (40, 41). Despite the small percentage of mutant cells, the clinical phenotype of mosaic patients is comparable with that of patients with germline mutations. Therefore, it remains controversial whether these low-frequency *NLRP3* mutant-positive cells alone are responsible for the disease phenotype or whether cells other than those harboring *NLRP3* mutations also contribute to pathogenesis.

Since each iPSC clone originates from a single cell (42), iPSC lines can be used as a discovery tool to evaluate the impact of low-frequency somatic mosaicism mutations. Taking advantage of this feature, *NLRP*3-mutant and wild-type (WT) iPSCs were established from patients with CINCA syndrome harboring a somatic *NLRP3* mutation (4). When these iPSCs were differentiated into macrophages and their phenotypes were compared, only *NLRP3*-mutant macrophages produced a large amount of IL-1β. Interestingly, when mutant macrophages were co-cultured with WT macrophages to create a pseudo-mosaic state, the production of IL-1β was significantly higher than that of mutant macrophages alone. In other words, in cases of somatic mosaicism, *NLRP3*-mutant cells are the main producers of IL-1β, although WT cells also contribute to inflammation in some way. Later, Baroja-Mazo et al. used patient-derived MDMs to show that upon activation of caspase-1, oligomeric NLRP3 inflammasome particles are released from activated macrophages and phagocytosed by surrounding macrophages, leading to further activation of caspase-1 (43). Thus, iPSCs can be used for a detailed analysis of the unique pathology associated with somatic mosaicism.

Regarding the pathogenesis of cartilage overgrowth in CINCA syndrome patients, different methods have been used to assess the contribution of chondrocytes and hematopoietic cells. After differentiating WT or mutant iPSCs into chondrocytes, we compared the size of the chondrocyte tissues produced; we found that mutant iPSCs produced larger chondrocyte masses than WT iPSCs owing to the overproduction of glycosaminoglycans, which correlated with increased expression of the chondrocyte master regulator SOX9; this was independent of caspase 1 and IL-1 and, thus, the NLRP3 inflammasome (5). By contrast, Wang et al. used a model mouse exhibiting global NLRP3 activation and several characteristics of the human disease (i.e., systemic inflammation and cartilage dysplasia) to show that activation of NLRP3 in myeloid cells, but not in mesenchymal cells, triggers chronic inflammation, which ultimately causes growth plate and epiphyseal dysplasia (44). Mechanistically, inflammation causes severe anemia and hypoxia in the bone environment but downregulates the HIF-1α pathway in chondrocytes, thereby promoting the demise of these cells. It is theoretically possible to obtain both PSC-derived chondrocytes and macrophages and evaluate their interaction *in vitro* co-cultures; however, mouse models may provide the opportunity to observe physiological interactions over a longer term than PSC-derived somatic cell models.

### Blau Syndrome

Blau syndrome (MIM 186580) is a disease caused by a heterozygous gain-of-function mutation in *NOD2* gene, which leads to granulomatous lesions in the skin, joints, and eyes during childhood and can cause severe complications such as blindness and joint contractures later in life (45, 46). The NOD2 protein is an intracellular pathogen recognition receptor, which upon recognition of the ligand MDP activates the nuclear factor-κB (NF-κB) pathway, thereby upregulating the production of proinflammatory cytokines and chemokines. Although pathological studies reveal that granulomas in Blau syndrome patients are accompanied by a prominent expression of IFN-γ (47), the details regarding the molecular mechanism(s) by which *NOD2* mutations drive the pathogenesis of Blau syndrome are unclear. The treatment for Blau syndrome has long been non-specific immunosuppressive therapies such as corticosteroids and/or methotrexate; however, recent studies report the effectiveness of biologics targeting TNF, IL-6, and IL-1 (48–53). Among these, anti-TNFα agents are used most widely, although the pharmacologic mechanism is unknown. Therefore, investigation of the cellular phenotypes of patients is necessary to evaluate the mechanism(s) underlying anti-TNFα therapy.

Studies based on mouse models have not reproduced the disease-related phenotype sufficiently (54). Therefore, we investigated the phenotypes of human macrophages carrying mutant *NOD2* by establishing iPSCs from patients with a *NOD2* mutation and obtaining isogenic iPSC clones in which the mutation was repaired by CRISPR/Cas9; we then differentiated them into macrophages (6). We found that IFN-γ-primed PSC-MPs harboring mutant *NOD2* demonstrated ligand-independent activation of NF-kB translocation to the nucleus, followed by the production of proinflammatory cytokines such as IL-6 and IL-8. We also confirmed this phenotype in more physiological cells; indeed, MDMs derived from Blau syndrome patients showed IFN-γ-induced ligand-independent activation of NF-kB translocation to the nucleus and subsequent cytokine production.

Next, we tried to clarify how anti-TNF treatment helps to control inflammation by comparing characteristics such as transcriptome profiling and cytokine secretion by MDMs from untreated and anti-TNF-treated Blau syndrome patients (7). We found that TNF-dependent NF-kB signaling reduces the threshold for IFN-γ-mediated inflammatory responses in Blau syndrome and that resetting of this primed state by anti-TNF treatment contributes to the prevention of the autoinflammatory loop, even in the presence of a *NOD2* mutation and IFN-γ stimulation. Thus, the iPSC-based macrophage study enabled us to elucidate disease pathogenesis and to identify the mechanism underlying the efficacy of anti-TNF treatment at the cellular level. To ascertain whether blocking IFN-γ signaling is a potential treatment for chronic inflammation in Blau syndrome patients, we still need to determine whether the IFN-γ pathway is actually activated in these patients and whether IFN-γ signaling is the principal priming pathway among the stimulants known to upregulate NOD2 expression (i.e., TNF-α, LPS, and other Toll-like receptor ligands) (55–57).

### Nakajo–Nishimura Syndrome

Nakajo–Nishimura syndrome (NNS)/chronic atypical neutrophilic dermatosis with lipodystrophy and elevated temperature (CANDLE) syndrome/joint contractures, muscular atrophy, microcytic anemia, and panniculitis-induced lipodystrophy (JMP) syndrome is a form of proteasome-associated autoinflammatory syndrome (PRAAS1/MIM 256040) characterized by chronic inflammation and lipomuscular atrophy caused by homozygous loss-of-function mutations in *PSMB8* gene encoding β5i, a component of the immunoproteasome (58, 59). Based on the finding of a strong type I interferon (IFN) response gene signature in patient peripheral blood cells (60, 61), Janus kinase (JAK) inhibitors are an effective treatment for PRAAS (62) because they inhibit the JAK/STAT pathway, the principal signaling pathway downstream of cytokines and growth factor receptors (including the IFN-α/β receptor) (63, 64). However, the precise mechanism underlying autoinflammation remains unclear. To elucidate the impact of *PSMB8* mutations on monocyte/macrophage function, we generated iPSCs from an NNS patient and repaired the PSMB8 mutation using the CRISPR/Cas9 system (8). We then generated iPSC lines that share the same genetic background but without the *PSMB8* mutation. When immunoproteasome assembly in PSC-MLs was induced by IFN-γ and TNF-α, immunoproteasome activity in *PSMB8*-mutant PSC-MLs was impaired significantly compared with that in the WT counterparts. As a consequence, secretion of the proinflammatory cytokine IL-6, and chemokines MCP-1 and IP-10, by mutant PSC-MLs increased. Furthermore, we revealed that the production of intracellular reactive oxygen species also increased, that mutant cells had higher levels of p38 MAPK and phosphorylated STAT1, and that addition of antioxidants, a p38 MAPK inhibitor, or JAK inhibitors suppressed the production of proinflammatory cytokines and chemokines. This demonstrates that PSC-MLs is a useful tool for modeling proteasome-associated autoinflammatory diseases.

Several different mechanisms have been postulated to explain the lipodystrophy in PRAAS. On the one hand, lipophagia can result from the proinflammatory state of adipose tissue macrophages (65, 66). Alternatively, high IFN levels may be toxic to adipocytes (67). PSC-derived blood cells alone cannot reproduce the complex interactions within the human body. Verhoeven et al. reported that hematopoietic stem cell transplantation halted the progression of lipodystrophy in a PRAAS patient during a 7-year follow-up, demonstrating that hematopoietic cells play a role in the lipodystrophy (68). It is theoretically possible to obtain both macrophages and adipocytes from PSCs and to evaluate their interaction in *in vitro* co-culture; however, to examine long-term effects, clinical observation of the patients may be more appropriate.

### Inflammatory Bowel Disease Caused by Loss of IL-10 Signaling

IL-10, one of the most important cytokines for the maintenance of intestinal homeostasis, regulates inflammation by inhibiting macrophage activation (69, 70). While the protective role of IL-10 is relatively well established in the context of inflammatory bowel disease (IBD) and other inflammatory diseases, its role in susceptibility to infections is less well understood. To study the impact of IL-10 on the inflammatory and microbicidal activities of macrophages, Mukhopadhyay et al. established iPSCs from a patient with homozygous loss-of-function mutations in the IL-10 receptor β (IL-10RB) and differentiated them into macrophages (9). They showed that IL-10RB−/− patient PSC-MPs were deficient in the IL-10 signaling pathway and that suppression of proinflammatory cytokine secretion was not observed upon simultaneous stimulation with IL-10 and LPS. IL-10RB−/− macrophages also exhibited a defect in bactericidal activity. Genes involved in synthesis and receptor pathways for PGE2 were more highly induced in IL-10RB−/− PSC-MPs, and these macrophages produced more PGE2 than controls after LPS stimulation. Furthermore, combined inhibition of PGE2 synthesis and receptor binding increased bactericidal activity. These results indicate the presence of crosstalk between the IL-10 and PGE2 pathways, dysregulation of which may drive aberrant macrophage activation and impaired host defense, thereby contributing to IBD pathogenesis.

## Functional Analysis to Facilitate a Correct Diagnosis

### Diagnosis of Somatic *NLRC4* Mosaicism in a Patient With Chronic Infantile Neurologic Cutaneous and Articular Syndrome

As mentioned above, while about 90% of CINCA syndrome patients have constitutive or somatic mosaic mutations in *NLRP3*, the remaining 10% do not (41). Since most CINCA patients lacking *NLRP3* mutations respond to anti-IL-1 therapy, activation of some kind of inflammasome is suspected. Therefore, we established iPSCs from a CINCA syndrome patient in whom an *NLRP3* mutation was not identified by conventional Sanger sequencing, differentiated them into PSC-MLs, and measured the production of IL-1β in response to NLRP3 inflammasome activation (10). PSC-ML clones were categorized as “normal” clones that secreted IL-1β after LPS and ATP stimulation and “pathological” clones that secreted IL-1β after LPS stimulation alone. To elucidate the phenotypic heterogeneity of IL-1β secretion among the clones, we performed whole-exome sequencing of representative iPSC clones and identified a novel mutation in *NLRC4* gene in the diseased clones. The mutant allele was observed in the patient’s fibroblasts (34.1%) and PBMCs (30.3%). When we knocked out the mutant *NLRC4* gene in PSC-MLs, the production of IL-1β normalized. These results show that somatic mosaicism of the *NLRC4* gene mutation caused the clinical phenotype of CINCA syndrome in this patient. To date, no other case of somatic mosaicism of *NLRC4* has been reported. Collectively, these data show that iPSC technology can be used to diagnose a novel somatic mosaic mutation.

### Diagnosis of Cell Type-Dependent Quantitative NF-κB Essential Modulator Deficiency Caused by a Deep Intronic Mutation

NF-κB essential modulator (NEMO), also known as an inhibitor of NF-κB kinase subunit gamma (IKK-γ), encoded by *IKBKG* gene (71, 72), is the third regulatory subunit of the IκB kinase (IKK) complex (73, 74). Amorphic mutations of *IKBKG*, which abolish canonical NF-κB activation, are lethal in men, whereas in women, they cause X-linked dominant incontinentia pigmenti (IP) (MIM 308300), a multisystem disorder affecting the skin and its appendages (75, 76). Hypomorphic *IKBKG* mutations that impair IκBα phosphorylation and sequential NF-κB activation cause X-linked recessive (XR) anhidrotic ectodermal dysplasia with immunodeficiency (EDA-ID) (MIM 300291) (77, 78). Affected men display typical signs of EDA, including sparse hair, eyebrows, and eyelashes; hypohidrosis; hypodontia; and conical incisors, together with immunodeficiency or inflammatory colitis (79). The main immune phenotype of EDA-ID is immunodeficiency rather than inflammation, but both EDA-ID and autoinflammatory diseases are categorized within the broad spectrum of primary immunodeficiency (80). Since iPSC-derived macrophages and ectodermal cells contributed substantially to establishing a correct diagnosis, we would like to describe the following study. While most variants underlying XR-EDA-ID are missense mutations or in-frame indels, approximately 10% of sporadic and familial cases of EDA-ID remain genetically unexplained. Therefore, we investigated three male patients from two families whose ID phenotype was much more severe than the manifestations of EDA (11), leading to an early death before the age of 1 year. Whole-genome sequencing identified the same deep intronic mutation in *IKBKG*. Next, we found that this deep intronic *IKBKG* mutation created a novel splicing donor site for a pseudoexon inclusion, which led to a severe decrease in NEMO protein expression and inflammatory cytokine secretion by patient PBMCs. Using patient-derived iPSCs, we revealed a cell type-dependent effect of the mutation on aberrant *IKBKG* splicing, which explains the reason for the discrepancy between the severe ID phenotype and the more subtle EDA symptoms. When we measured the levels of WT and alternative *IKBKG* transcripts in undifferentiated iPSCs, PSC-MLs, and iPSC-derived neuronal precursor cells (iPSC-NPs), we found that iPSCs produced 17% WT transcripts, PSC-MLs produced only 3% WT transcripts, and iPSC-NPs produced 35%. Patient-derived PSC-MLs showed much lower WT NEMO protein levels, along with impaired NF-κB activation upon LPS stimulation. Complementation of patient-iPSCs with WT NEMO restored NF-κB pathway activation in PSC-MLs. Thus, iPSCs contribute to the correct diagnosis of the deep intronic *IKBKG* mutation and to the identification of the cell type-dependent quantitative NEMO deficiency, thereby expanding our understanding of this disease.

### Functional Evaluation of the Pathological Significance of *MEFV* Variants

Monocytes and macrophages play similar roles in the pathogenesis of most inflammatory diseases. For example, both *NLRP3*-mutant monocytes (37) and macrophages (4) exhibit spontaneous NLRP3 inflammasome activation without secondary signals and secrete IL-1β after priming with LPS alone. However, cytokine secretion by monocytes and macrophages from familial Mediterranean fever (FMF) patients is clearly different (12).

FMF, the most common hereditary autoinflammatory disorder, is characterized by recurrent episodes of fever, polyserositis, and abdominal pain (MIM 249100). FMF is associated with mutations in *MEFV* gene, which encodes the inflammasome adaptor pyrin (81). Pyrin is an inflammasome sensor that detects imbalances in Rho GTPase activity, which can be caused by bacterial effectors or bacterial toxins (82). Almost 400 *MEFV* variants have been recorded in *Infevers*, an online database of autoinflammatory disease mutations. Among *MEFV* variants, a systematic review revealed that M694V and M694I in exon 10 are related to a severe phenotype of FMF (83). Other *MEFV* variants are associated with variable clinical phenotypes, including pyrin-associated autoinflammation with neutrophilic dermatosis (84, 85) and autosomal-dominant FMF-like diseases (86–88). Consequently, the novel umbrella term, pyrin-associated autoinflammatory diseases, has been proposed to define all autoinflammatory diseases caused by *MEFV* mutations (89). Although a consensus-driven pathogenicity classification was proposed recently to support the uniform diagnosis of FMF worldwide (90), the complexity of the clinical phenotype and its association with *MEFV* variants led to difficulty in assessing the pathogenicity of variants identified in clinical settings. Despite the successful use of colchicine and IL-1β-blocking therapies as treatments for FMF, *in vitro* pyrin inflammasome activation (and its inhibition by colchicine) in patients’ hematopoietic cells remains controversial (91–94).

To clarify this issue, we evaluated cytokine secretion by primary monocytes and MDMs obtained from FMF patients carrying the heterozygous M694I mutation. In response to TcdA stimulation, levels of IL-1β secreted by FMF monocytes were similar to those of control monocytes, and colchicine failed to inhibit IL-1β secretion by FMF monocytes. By contrast, IL-1β secretion by FMF MDMs was significantly higher than that by control MDMs in response to LPS and TcdA, and IL-1β secretion by FMF MDMs was inhibited by colchicine. These results suggest that MDMs, rather than monocytes, reflect the clinical features of FMF patients (e.g., hyperactivation of the pyrin inflammasome and subsequent inhibition by colchicine). After confirming that macrophages derived from patients’ iPSCs (PSC-MPs) recapitulate the phenotype of FMF MDMs, we evaluated two rare *MEFV* variants, T577N and N679H, identified in two families in which autoinflammatory disease with dominant inheritance was suspected (95, 96). No T577N patients met the Tel-Hashomer criteria, whereas two N679H patients fulfilled the criteria (97). Whereas the amount of IL-1β secreted by T577N iPS-MPs was comparable with that secreted by WT cells, N679H iPS-MPs secreted significantly more IL-1β (like the M694I variant). Thus, *MEFV* variants causing FMF, namely, N679H and M694I, induced IL-1β secretion after pyrin inflammasome activation. Thus, we established a method for evaluating *MEFV* variants by obtaining mutant PSC-MPs and measuring cytokine secretion in response to pyrin inflammasome stimulation (12).

In addition, we characterized cytokine secretion by primary monocytes and macrophages isolated from typical FMF patients. Gene expression differs considerably between monocytes and macrophages, including expression of the tubulin-related genes (98). Given the vital role of microtubule polymerization in pyrin inflammasome activation (99), we speculate that greater expression of tubulin-related genes in macrophages might be related to the differential response to colchicine inhibition between monocytes and macrophages. Moreover, monocytes and macrophages are somewhat different in terms of inflammasome activation pathways. For example, although both cell types use the canonical NLRP3 inflammasome activation pathway, the alternative (100) or non-canonical (101) NLRP3 inflammasome pathway is functional only in monocytes. It is possible that an undiscovered pyrin inflammasome activation pathway is functioning in either monocytes or macrophages, but not in both. The precise reason underlying the distinct mechanisms of pyrin inflammasome activation in these cells remains to be elucidated.

Evaluation of pyrin inflammasome activation using PSC-MPs led to the discovery of a role for enhanced pyrin inflammasome activation in the pathogenesis of CDC42-associated autoinflammation (102). Nishitani-Isa et al. used PSC-MPs to show that aberrant palmitoylation of CDC42 protein carrying the mutation caused its retention in the Golgi apparatus and triggered overactivation of the pyrin inflammasome. By contrast, subsequent *ex vivo* or *in vitro* studies established methods for functional evaluation of FMF-related *MEFV* variants. THP-1 cells transfected with FMF-related *MEFV* variants showed higher levels of UCN-01/TcdA-induced cell death than THP-1 cells expressing other *MEFV* variants (103). Similar studies focusing on the evaluation of primary cells have been reported; indeed, Magnotti et al. showed that UCN-01-induced cell death was much faster in FMF monocytes than in monocytes from healthy donors or patients suffering from other inflammatory disorders. They also established an assay that can be used for rapid diagnosis of FMF with high sensitivity and specificity (104). By focusing on unresponsiveness to colchicine inhibition, van Gorp et al. reported an assay that robustly segregated FMF patients from healthy donors and patients with other inflammatory disorders (105). One advantage of this assay is that the test may be performed on PBMCs and even whole blood. While cell line-based approaches are free from the influence of existing inflammation or prescribed drugs, as well as being more suitable for the evaluation of a specific mutation, primary cell assays have an advantage in that they take less time and the results reflect the influence of the genetic background, or the combined effects of multiple *MEFV* variants, in a single patient. Therefore, we need to select the most appropriate approach for each situation.

## Evaluation of the Disease Relevancies of Novel Mutations

### Autoinflammatory Immunodeficiency Caused by Heterozygous *OAS1* Gain-of-Function Variants

Type I IFN-inducible oligoadenylate synthetase 1 (OAS1) initiates an antiviral immune response upon recognition of cytoplasmic viral double-stranded RNA (dsRNA) (106, 107). OAS1 is a template-independent nucleotidyltransferase that produces the second messengers 2′-5′-oligoadenylate (2-5A) (108, 109). In turn, 2-5A activates ribonuclease L (RNase L), which degrades viral and cellular RNA, thereby interfering with viral propagation (110).

Okano et al. described a polymorphic autoinflammatory immunodeficiency with recurrent fever, dermatitis, IBD, PAP, and hypogammaglobulinemia caused by *de novo* heterozygous OAS1 gain-of-function mutations; they named the disease OAS1-associated polymorphic autoinflammatory immunodeficiency (OPAID) (13). The expression of mutant OAS1 in response to common infectious agents resulted in an inappropriate synthesis of 2-5A independent of dsRNA binding; this induced RNase L-mediated cleavage of cellular RNA, leading to transcriptomic alteration, translational arrest, and dysfunction and apoptosis of primary monocytes, PSC-MPs, and B cells. To overcome the scarcity of primary monocytes in that study, the authors differentiated patient-derived iPSCs into macrophages (i.e., PSC-MPs). IFNα-stimulated PSC-MPs carrying mutant OAS1 displayed impaired cell adhesion and clustering, scavenger receptor expression, and phagocytosis in an RNase L-dependent manner. While mutant OAS1-knock-in mice failed to reproduce the disease phenotype (111), the characteristics of PSC-MPs were consistent with those of primary monocytes. Given the reported differences in immune responses between species (112), human iPSC-derived hematopoietic cells may be a more relevant source of primary patient-derived cells than animal models for research into certain diseases.

### Paradoxical Autoinflammation Caused by a Dominant-Negative *NFKB1A* Mutation

The NF-κB protein complex is integral to the initiation of inflammation, and NF-κB activation is controlled by inhibitors of κB (IκBα, IκBβ, and IκBϵ) and by the IκB kinase (IKK) complex, which comprises NEMO, IKK1, and IKK2 (described above). Patients with genetic defects (e.g., in *NEMO* and *NFKBIA*) in the NF-κB signaling pathway usually display severe immunodeficiency, with impaired cellular responses to immune stimuli such as LPS or TNF-α (113, 114).

Tan et al. reported an infant with a clinical pathology comprising neutrophil-mediated autoinflammation and recurrent bacterial infections caused by a *de novo* heterozygous missense mutation in *NFKBIA* (14). The resulting L34P IκBα variant caused a severe reduction in NF-κB nuclear translocation and, consequently, downstream production of IL-6 or IL-8 by the patient’s fibroblasts. Paradoxically, IL-1β concentrations in the patient’s blood were elevated. To determine whether myeloid cells were the major source of elevated IL-1β levels, they generated iPSC-derived macrophages from the patient’s fibroblasts. Despite the patient’s PSC-MPs showing defective nuclear translocation of NF-κB in response to LPS stimulation, they produced significantly more IL-1β than control PSC-MPs. The patient’s hypersecretion of IL-1β correlated with activated neutrophilia and liver fibrosis with neutrophil accumulation. Hematopoietic stem cell transplantation reversed the neutrophilia, restored neutrophils to a resting state, and normalized IL-1β release from stimulated leukocytes. These data suggest that NF-κB in humans plays an unexpected role as an anti-inflammatory agent by regulating IL-1β secretion, thereby preventing myeloid inflammation.

## Discovery of Drug Candidates

Why are iPSC-derived monocytes/macrophages a useful platform for high-throughput screening of drug candidates? Because of their pluripotency and proliferative potential, iPSCs can serve as an unlimited source of patient-derived somatic cells. Two studies provide proof of the concept that iPSC-derived monocytes/macrophages are a useful tool for drug screening (15, 16).

In one study, we searched for compounds that inhibit NLRP3 inflammasome activation in PSC-MPs (15). The NLPR3 inflammasome is an attractive drug target because NLRP3 inflammasome activation is associated not only with rare autoinflammatory disorders such as CINCA syndrome but also with the pathogenesis of various chronic inflammatory conditions (115). *NLRP3*-mutant macrophages were used for this assay because LPS-mediated stimulation in the absence of a second signal was sufficient to activate the NLRP3 inflammasome (4). High-throughput screening of 4,825 compounds, including Food and Drug Administration (FDA)-approved drugs and compounds with known bioactivity, identified seven that selectively inhibited IL-1β secretion without affecting IL-6 production. All seven compounds inhibit the NLRP3 inflammasome (116–119). Before selecting the cell types for high-throughput screening, we compared three types of PSC-derived blood cells in terms of the coefficient of variation (CV) for IL-1β secretion; this is because blood cells with a low CV value enabled us to accurately assess the potency of candidate compounds. The first type of PSC-derived floating cells was obtained after a 2-week culture of iPSCs in a differentiation medium. The second cell type of PSC-MLs was established by the lentiviral-based introduction of three genes into PSC-derived floating cells (6, 10). After a 1-week culture of PSC-MLs in a fetal calf serum (FCS)-containing medium in the presence of M-CSF, we obtained a third type of terminally differentiated PSC-MP (**Figure 1**). The levels of released cytokines were more consistent, and the CV value became lower as differentiation progressed; the CV value was lowest for PSC-MPs. Therefore, we used PSC-MPs to screen NLRP3 inhibitors (15).

In another study, we screened potential therapeutic candidates using PSC-MLs derived from NNS patients (16); screening was based on consistent overproduction of MCP-1 and IL-10 from PSC-MLs derived from NNS patients in the preceding study (8). We screened 5,821 compounds, including FDA-approved drugs, kinase inhibitors, and bioactive chemicals, and we identified CUDC-907 as an effective inhibitor of MCP-1 and IP-10 release (16). While hit compound CUDC-907 seemed a promising drug candidate in terms of efficacy, there were concerns regarding adverse effects because CUDC-907 inhibited NNS fibroblast proliferation during a 2-week culture. Therefore, CUDC-907 was not directly applicable to the clinical study.

In both studies, we started screening compound libraries with known bioactivity and provided proof of concept that PSC-derived monocytes/macrophages can serve as an effective tool for screening drug candidates, although the hit compounds could not be applied directly to clinical studies. Combining high-throughput screening using PSC-monocytes/macrophages with pharmaceutical techniques (to generate a more potent compound from known substances by modifying the chemical structure) may pave the way to novel drug discovery.

## Limitations of These Approaches

The PSC–macrophage system would not necessarily be suitable for modeling all autoinflammatory diseases. Therefore, we would like to mention two points that should be considered before starting a study using PSC-MPs.

### Elucidation of the Pathologic Interaction Between Hematopoietic and Non-Hematopoietic Cells

PSC-derived macrophages are a homogeneous population, and modeling the whole human body using only macrophages is impossible. For example, RELA (120) or RIPK 1 (121) mutations were identified among patients with early-onset inflammatory diseases. Both proteins are involved in the NF-κB activation pathway in response to TNF stimulation (122). Since they noted enhanced cytotoxicity caused by TNF stimulation in fibroblasts derived from the RELA-haploinsufficiency patient (but not in hematopoietic cells) (120), PSC-MPs alone would be insufficient for modeling such diseases; rather, studies of the interaction among epithelial cells, stromal cells, and hematopoietic cells would be necessary. As described above, clinical observation provided novel findings of lipodystrophy in a PRAAS patient (68), and a knock-in mouse model revealed the contribution of *NLRP3*-mutant hematopoietic cells to cartilage overgrowth (44). Thus, elucidation of the pathologic interactions between hematopoietic and non-hematopoietic cells is usually difficult when using PSC-derived somatic cells alone.

### Similarities and Differences Between Monocyte-Derived Macrophages and Pluripotent Stem Cell-Derived Macrophages

Many researchers have identified similarities between MDMs and PSC-MPs with regard to global gene expression (23, 24, 26, 30, 32, 33), cytokine secretion (26), and phagocytosis of infectious organisms (32). Polarization of macrophages from M0 to M1 or M2 is accompanied by changes in gene expression similar to those observed in blood-derived counterparts (26, 33). However, certain differences in the expression of genes involved in chemokine production, antigen presentation, and tissue remodeling were identified (30). We need to be cautious when applying PSC-MPs for disease modeling because these differences may affect their responses.

## Future Directions

The list of autoinflammatory diseases continues to expand and now includes over 40 genetically defined disorders categorized according to defects affecting the inflammasome, type 1 interferonopathies, and non-inflammasome-related conditions (123). Although the genetic basis of many autoinflammatory diseases is now known, the molecular etiology frequently remains unclear. Given the rapid progress in the application of PSC-MPs to research into autoinflammatory diseases, further discoveries are expected. Takata et al. reported differentiation of PSCs into tissue-resident macrophage-like cells upon receipt of organ-specific cues (24). Co-culturing human PSC-MPs with iPSC-derived neurons *in vitro* promoted differentiation into microglia-like cells. Furthermore, murine PSC-MPs differentiated *in vivo* into functional alveolar macrophages after engraftment in the lung. Novel methods of driving differentiation into tissue-resident macrophages will enable modeling and elucidation of organ-specific inflammation. In addition to monocytes/macrophages, neutrophils are important innate immune cells that are involved in the pathogenesis of autoinflammatory disorders. While it is now possible to cryopreserve PSC-MPs at the progenitor level (6, 10), or as the final product (34), cryopreservation of PSC-derived neutrophils has yet to be reported. Improvements in the differentiation protocol may enable the utilization of PSC-derived neutrophils for autoinflammatory disease research. We would like to describe two other promising examples of PSC-MP applications.

### Drug Screening to Identify Alternative Pyrin Inflammasome Inhibitors

The scalability and standardizability of PSC-MPs make them particularly suitable for screening drug candidates. In addition to the diseases described above (15, 16), PSC-MPs may be useful for identifying pyrin inflammasome inhibitors other than the traditional colchicine. Colchicine has been the standard treatment for FMF for more than 100 years. However, in 5%–10% of patients, it is either ineffective or associated with unacceptable side effects (124). The efficacy and safety of canakinumab, an anti-IL-1β monoclonal antibody, have been shown in patients with colchicine-resistant FMF (125). However, given the drawbacks of canakinumab, such as high cost and limited safety information about administration to pregnant women, novel alternative treatments are needed. Recent reports about the association between microtubule polymerization and pyrin inflammasome activation (99, 126) have focused attention on microtubule inhibitors. Microtubules are the target of anticancer drugs such as paclitaxel or vinblastine. Potent and safe colchicine binding-site inhibitors such as CA4P and ABT-751 have entered clinical trials as anticancer agents (127). Although many other candidates have not entered clinical trials due to toxicity at the high concentrations needed for anticancer treatment, they may be effective and safe pyrin inflammasome inhibitors when used at lower concentrations. Among the tubulin inhibitors that were previously developed as anticancer medicines, we may be able to identify a more potent and less toxic pyrin inflammasome inhibitor than conventional colchicine.

### Investigation of the Interaction Between Innate and Adaptive Immunity in Patients With Interferonopathies

Originally, “autoinflammatory disorders” covered inborn errors of innate immunity (1). Classification of autoinflammatory diseases has been updated periodically and now covers not only abnormalities in innate immunity but also those in adaptive immunity, including STING-associated vasculopathy with onset in infancy (SAVI) or COPA syndrome. A severe inflammatory phenotype of SAVI is induced by gain-of-function mutations in *STING1*, which encodes STING (stimulator of IFN genes); this phenotype is characterized clinically by skin vasculopathy, systemic inflammation, and lung involvement (e.g., interstitial lung disease or diffuse alveolar hemorrhage), which is associated with high morbidity and mortality (128, 129). In addition to constitutive activation of the type I IFN pathway, an autoimmune component (e.g., high titers of autoantibodies) is frequently detected in SAVI (130). Self-DNA sensing through cGAS-STING is involved in many processes, including autoimmunity (131); however, the precise mechanism linking STING gain of function to the production of autoantibodies has not yet been defined.

While various groups have established methods for obtaining innate immune macrophages from PSCs (as described above), differentiating PSCs into T cells is difficult. However, a recent report describes a protocol for differentiating PSCs to T-cell receptor (TCR)-expressing innate lymphoid-like helper cells (132). These innate lymphoid helper-like cells induce bcr-abl-specific TCR signaling, which mediates effective anti-leukemic cytotoxic T lymphocyte responses *via* dendritic cell (DC) maturation. Further deciphering of STING-mediated autoimmunity is awaited, and further investigations into PSC-derived macrophages and T cells may provide an opportunity to study aberrant interactions between innate and adaptive immune cells.

## Conclusions

In this review, we provide a comprehensive overview of current advances in the use of iPSC-derived monocytes/macrophages for research into autoinflammatory diseases. We describe the advantages and characteristics of PSC-MPs, current applications to research into autoinflammatory diseases, and future directions. We hope that this review will provide clues that facilitate further research into autoinflammatory diseases and contribute to the development of new treatments for patients.

## Author Contributions

This article is mainly written by TT. YH wrote part of the manuscript. TS, KI, TY, and MS proofread the manuscript. RN revised the manuscript. The corresponding author had final responsibility for the decision to submit for publication. All authors contributed to the article and approved the submitted version.

## Funding

This research was supported by the following grant: Grants-in-Aids for Scientific Research (C) (grant JP19K08320 to T.T.).

## Conflict of Interest

The authors declare that the research was conducted in the absence of any commercial or financial relationships that could be construed as a potential conflict of interest.

## Publisher’s Note

All claims expressed in this article are solely those of the authors and do not necessarily represent those of their affiliated organizations, or those of the publisher, the editors and the reviewers. Any product that may be evaluated in this article, or claim that may be made by its manufacturer, is not guaranteed or endorsed by the publisher.
